# Supplementary material for: Architecture of marine food webs: To be or not be a ‘small-world’
Source: PLoS One. 2018 May 29;13(5):e0198217. doi: 10.1371/journal.pone.0198217 (PMC5973612; doi:10.1371/journal.pone.0198217)
Supplement: S1 Table — (DOCX) [file pone.0198217.s001.docx]

# Supporting information

**S1 Table.** **Comparison of the small-world-ness metric proposed by Humphries and Gurney (2008) (S^ws^) and our method (SW).**

S^ws^CI = confidence interval 99% for S^ws^. CPLe and CPL_CI = Characteristic Path Length for empiric and confidence interval 99% for random networks; CCe and CC_CI = Clustering Coefficient for empiric and confidence interval 99% for random networks, respectively. SWness = SW networks according to S^ws^; SW = SW networks according to our method.

| **Network** | **S^ws^** | **S^ws^CI** | **CPLe ≤ CPL_CI** | **CCe > CC_CI** | **SWness** | **SW** |
| --- | --- | --- | --- | --- | --- | --- |
| **La Guajira** | 1.103 | 1.055 | FALSE | TRUE | TRUE | FALSE |
| **Benguela** | 0.564 | 1.035 | FALSE | FALSE | FALSE | FALSE |
| **NE US Shelf** | 0.654 | 1.010 | FALSE | FALSE | FALSE | FALSE |
| **Gulf of Cadiz** | 0.903 | 1.036 | FALSE | TRUE | FALSE | FALSE |
| **Baltic Sea** | 1.008 | 1.113 | TRUE | FALSE | FALSE | FALSE |
| **Beagle Channel** | 1.063 | 1.127 | TRUE | FALSE | FALSE | FALSE |
| **Angola** | 1.137 | 1.181 | TRUE | FALSE | FALSE | FALSE |
| **Chilean rocky** | 0.590 | 1.022 | TRUE | FALSE | FALSE | FALSE |
| **Gulf of Lions** | 1.375 | 1.227 | TRUE | TRUE | TRUE | TRUE |
| **Florida** | 1.756 | 1.319 | TRUE | TRUE | TRUE | TRUE |
| **Simon Bay** | 1.040 | 1.788 | TRUE | FALSE | FALSE | FALSE |
| **Celtic Sea** | 1.896 | 1.551 | FALSE | TRUE | TRUE | FALSE |
| **Cuba** | 0.822 | 1.026 | TRUE | FALSE | FALSE | FALSE |
| **Jamaica** | 0.921 | 1.027 | TRUE | FALSE | FALSE | FALSE |
| **Cayman Is.** | 0.868 | 1.026 | TRUE | FALSE | FALSE | FALSE |
| **Monterey Bay** | 1.421 | 2.241 | TRUE | FALSE | FALSE | FALSE |
| **Barents Sea Boreal** | 2.380 | 1.185 | FALSE | TRUE | TRUE | FALSE |
| **Caribbean reef (l)** | 1.526 | 1.064 | TRUE | TRUE | TRUE | TRUE |
| **Potter Cove** | 1.684 | 1.607 | TRUE | FALSE | TRUE | FALSE |
| **Southern Brazil** | 0.552 | 1.073 | FALSE | FALSE | FALSE | FALSE |
| **Barents Sea Arctic** | 2.757 | 1.474 | TRUE | TRUE | TRUE | TRUE |
| **Beach Peru** | 2.372 | 6.946 | TRUE | FALSE | FALSE | FALSE |
| **Sanak intertidal** | 1.703 | 1.168 | FALSE | TRUE | TRUE | FALSE |
| **Sanak nearshore** | 2.242 | 1.095 | FALSE | TRUE | TRUE | FALSE |
| **SW Pacific Ocean** | 1.806 | 4.386 | TRUE | FALSE | FALSE | FALSE |
| **Gulf of Alaska** | 0.343 | 1.221 | TRUE | FALSE | FALSE | FALSE |
| **Gulf of Tortugas** | 2.236 | 2.610 | TRUE | FALSE | FALSE | FALSE |
| **Weddell Sea** | 3.033 | 1.714 | TRUE | TRUE | TRUE | TRUE |
